# Transcriptome Analysis Unveils the Molecular Mechanisms of Ethylene-Induced Ready-to-Eat Kiwifruit-Picking Ripening

**DOI:** 10.3390/foods14122026

**Published:** 2025-06-08

**Authors:** Jiayi Zhang, Shangqiao Cao, Na Li, Hongbo Li, Zhenbin Liu, Dan Xu, Haizhen Mo

**Affiliations:** 1 School of Food and Biological Engineering, Shaanxi University of Science and Technology, Xi’an 710021, China; 2 Shaanxi Agricultural Products Processing Technology Research Institute, Xi’an 710021, China; 3 Institute of Basic and Translational Medicine, Xi’an Medical University, Xi’an 710021, China

**Keywords:** transcriptomic analysis, ripening mechanism, ready-to-eat kiwifruit, fruit softening, aroma biosynthesis, ethylene

## Abstract

Kiwifruit is a climacteric fruit that undergoes significant physiological and biochemical changes during ripening, with ethylene playing a central regulatory role. Understanding the molecular mechanisms underlying ethylene-induced ripening is crucial for improving the postharvest handling and quality of ready-to-eat kiwifruit. The primary objective of the present study was to comprehensively analyze the transcriptome to investigate the ripening mechanism of ethylene-induced ready-to-eat kiwifruit. During the rapid maturation phase, it was observed that the gene *Acc26812*, responsible for regulating malate synthase activity, showed a significant upregulation at 84 h. Similarly, the gene *Acc07097*, which encodes arginine decarboxylase, also showed a significant upregulation during this period. A canonical correlation analysis (CCA) was performed to ascertain the relevance of genes associated with fruit firmness. Through transcriptome sequencing and bioinformatics analysis, approximately 2000 differentially expressed genes (DEGs) were identified. These genes were primarily involved in various pathways such as pentose and glucuronic acid interconversion, DNA replication, and others. A further investigation of these DEGs provided insights into several biological processes and molecular activities that contribute to the regulation of kiwifruit firmness. Notably, genes associated with fruit softening, including pectinesterase and cellulase, demonstrated significant upregulation, thereby indicating the degradation and remodeling of cell wall components during ripening. Additionally, highly expressed genes involved in glucose synthesis and transport highlighted the crucial role of sugar synthesis in the maturation process of ready-to-eat kiwifruit. Consequently, this study offers valuable insights into the mechanisms underlying the maturation of ready-to-eat kiwifruit.

## 1. Introduction

Kiwifruit (*Actinidia Chinensis*) is a globally cultivated crop with increasing economic significance due to its high nutritional value and versatile culinary applications [1]. Originally native to China, it was introduced to New Zealand in 1904, where it became a commercially important fruit crop before being reintroduced to China in the 1970s [2]. Today, China dominates global kiwifruit production, with an annual output of 1.06 million tons (38% of global production) and a cultivation area of 180,000 hectares (59% of global acreage) [3,4]. Shaanxi Province alone contributes 61% of China’s production, serving as the primary hub for both fresh consumption and processed products such as juice, vinegar, dried slices, jam, wine, yogurt, and jelly. Rich in vitamin C, fiber, potassium, and antioxidants, kiwifruit has been associated with numerous health benefits, including reducing the risk of cardiovascular diseases, cancer, and diabetes [5]. However, despite its economic and nutritional potential, kiwifruit faces challenges related to postharvest handling, storage, transportation, and consumer preferences for specific taste, texture, and appearance [6,7]. The ripening process plays a critical role in determining the quality, yield, and marketability of kiwifruit, involving complex physiological, biochemical, and molecular changes [8]. Therefore, understanding the molecular mechanisms and regulatory networks underlying kiwifruit ripening is essential for enhancing postharvest performance and meeting consumer demands.

Ethylene accelerates the ripening and softening in fruits, particularly climacteric varieties, through the coordinated regulation of its biosynthesis, spatial distribution, signal transduction, and downstream gene expression [9]. Molecular analyses of ethylene-treated fruits including tomato, melon, kiwifruit, strawberry, avocado, and apricot demonstrate that the ethylene-mediated upregulation of cell-wall-modifying (CWM) genes such as PG and PL constitutes a conserved mechanism driving fruit softening [10,11,12].

The ripening process in kiwifruit involves various physiological, biochemical, and molecular changes. The distinct stages include the pre-climacteric, climacteric, and post-climacteric stages. During the pre-climacteric stage, kiwifruits are considered unripe and firm, and exhibit low respiration rates. As kiwifruits enter the climacteric stage, there is a sharp increase in respiration rates, ethylene production, fruit softening, color changes, and aroma development [13]. In the post-climacteric stage, respiration rates, ethylene production, and fruit quality decline. However, the regulatory mechanisms underlying kiwifruit ripening remain complex and not fully elucidated. Comparative studies with other fruits, such as bananas, can shed light on the variability and diversity of ripening mechanisms across different plant species [14,15].

Previous research has revealed significant differences in the regulation of ripening mechanisms among various fruits. For example, tomato ripening primarily relies on a single gene called the ripening inhibitor (*RIN*), encoding a transcription factor that positively regulates ripening-related gene expression [16]. In contrast, banana ripening involves a complex network of transcription factors, signaling pathways, and epigenetic modifications [17,18]. Similarly, the regulation of kiwifruit ripening likely entails a diverse set of genes and pathways [19,20,21]. Therefore, understanding the regulatory network underlying rapid ripening in kiwifruit could provide valuable insights into the ripening mechanisms of other fruits and aid in developing strategies to enhance kiwifruit’s postharvest performance.

Previous studies employing transcriptomic, proteomic, and genetic analyses have identified the key gene families and regulatory pathways involved in kiwifruit ripening. The transcriptome analysis has demonstrated significant changes in the expression of genes related to ethylene biosynthesis, cell wall modification, and sugar metabolism during kiwifruit ripening [21,22,23]. Proteomic studies have identified differentially expressed proteins associated with organic acid and amino acid metabolism, as well as antioxidant defense during ripening [24]. Furthermore, genetic investigations have highlighted the involvement of transcription factor families, such as MYB and NAC, in regulating kiwifruit ripening [25,26].

Despite these advancements, our understanding of the regulatory network governing rapid ripening in kiwifruit remains incomplete, particularly for commercially pivotal cultivars like Actinidia deliciosa cv. Xuxiang. As one of China’s most widely planted and economically important kiwifruit varieties, Xuxiang exhibits distinct ripening characteristics and postharvest challenges that differ from other cultivars; yet, its molecular mechanisms remain underexplored. In addition, prior works focused on differential gene screening, while our CCA-based approach is the first to establish a multivariate gene-quality regulatory network, which is critical for understanding systemic maturation control. Therefore, this study aims to explore the regulatory mechanisms of ripening-related molecules through transcriptome sequencing, providing a foundation for the development of new biological agents for ready-to-eat kiwifruit (Actinidia deliciosa cv. Xuxiang) ripening.

## 2. Materials and Methods

### 2.1. Plant Material and Ethylene Treatment

Kiwifruit samples of cultivar Xuxiang were harvested at commercial maturity (170 days post-bloom, before 10 a.m.) from an orchard in Baoji, Shaanxi Province, China, and transported to the laboratory. After a 24 h callus treatment to dissipate field heat, fruits were divided into three groups (96 fruits/group): two groups were treated with 1000 μL L⁻^1^ ethylene, while the third served as an untreated control. Fruits were stored in sealed plastic boxes (60 L, 52.5 × 40 × 28.5 cm) at 25 °C, with physiological parameters (e.g., firmness, soluble solids content (SSC), pH, L*, a*, b*, respiration rate, weight loss rate, and total soluble sugar (TSS)) monitored every 12 h over 84 h [27,28]. Three biological replicates were analyzed. Flesh samples were flash-frozen in liquid nitrogen and stored at −80 °C for transcriptomic analysis.

### 2.2. Sequence and Filtering of Clean Reads

A cDNA library was created using pooled RNA from the described research species samples and subsequently sequenced using the Illumina Novaseq ^TM^ 6000 sequencing platform [29]. The transcriptome was sequenced utilizing the Illumina paired-end RNA-seq approach, resulting in a total of million 2 *×* 150 bp paired-end reads. During the sequencing process, the obtained reads included raw reads containing adapters or low-quality bases that could potentially impact subsequent assembly and analysis. To ensure the acquisition of high-quality clean reads, further filtering of the reads was performed using Cutadapt.

### 2.3. Transcriptome Sequencing

Samples were subjected to regular sequencing every 12 h throughout the 84 h ripening process. Transcriptome sequencing was performed using three replicates. Total RNA was extracted using Trizol reagent (Thermo Fisher, Waltham, MA, USA, 15596018), and the quality and quantity of RNA were assessed using the Bioanalyzer 2100 and RNA 6000 Nano LabChip Kit (Agilent, Folsom, CA, USA, 5067-1511). High-quality RNA samples with an RIN number > 7.0 were selected for library construction. After total RNA extraction, mRNA was purified from 5 µg of total RNA using Dynabeads Oligo (dT) (Thermo Fisher, CA, USA) through two rounds of purification. The purified mRNA was fragmented into short fragments using divalent cations under elevated temperature (Magnesium RNA Fragmentation Module, NEB, cat. e6150, Ipswich, MA, USA) at 94 °C for 5–7 min. The resulting RNA fragments were reverse-transcribed using SuperScript™ II Reverse Transcriptase (Invitrogen, cat. 1896649, Carlsbad, CA, USA) to generate cDNA. The cDNA was then used to synthesize U-labeled second-stranded DNAs with *E. coli* DNA polymerase I (NEB, cat.m0209, Ipswich, MA, USA), RNase H (NEB, cat.m0297, MA, USA), and deoxyuridine triphosphate (dUTP) Solution (Thermo Fisher, cat. R0133, Vacaville, CA, USA) [30]. An A-base was added to the blunt ends of each strand to facilitate ligation to the indexed adapters. Each adapter contained a T-base overhang for ligating the adapter to the A-tailed fragmented DNA. Dual-index adapters were ligated to the fragments. fSize selection was performed using AMPureXP beads. Subsequently, the U-labeled second-stranded DNAs were treated with heat-labile UDG enzyme (NEB, cat.m0280, MA, USA), followed by PCR amplification under the following conditions: initial denaturation at 95 °C for 3 min; 8 cycles of denaturation at 98 °C for 15 s, annealing at 60 °C for 15 s, and extension at 72 °C for 30 s; and a final extension at 72 °C for 5 min. The average insert size for the final cDNA libraries was 300 ± 50 bp. Finally, paired-end sequencing (PE150) was performed on an Illumina Novaseq™ 6000 (LC-Bio Technology CO., Ltd., Hangzhou, China).

### 2.4. Differentially Expressed Genes Analysis

Differential expression analysis of genes was conducted using DESeq2 software (1.48.0) for comparisons between two distinct groups, and edgeR was utilized for comparisons between two samples. Genes meeting the criteria of a false discovery rate (FDR) below 0.05 and an absolute fold change ≥ 2 were categorized as differentially expressed genes (DEGs). Subsequently, DEGs underwent enrichment analysis to identify enriched Gene Ontology (GO) functions and Kyoto Encyclopedia of Genes and Genomes (KEGG) pathways.

### 2.5. Gene Ontology Enrichment Analysis

*Gene Ontology Enrichment Analysis* (GO) is an internationally standardized gene functional classification system that provides a dynamic and regularly updated controlled vocabulary. GO enrichment analysis aims to identify GO terms that exhibit significant enrichment among DEGs compared to the background genome.

In this study, DEGs were initially mapped to corresponding GO terms within the Gene Ontology database. The number of genes associated with each term was calculated, and hypergeometric tests were employed to determine significantly enriched GO terms among the DEGs relative to the genome background. The parameters used in this context are as follows: N represents the total number of genes with GO annotation (referred to as total background gene or TB gene number); n corresponds to the number of DEGs in N (total significant gene or TS gene number); M represents the total number of genes annotated to specific GO terms (background gene or B gene number); and m signifies the number of DEGs in M (significant gene or S gene number). Significantly enriched GO terms were identified based on the condition of *p* < 0.05. This analysis was able to recognize the main biological functions that DEGs exercise.

### 2.6. Kyoto Encyclopedia of Genes and Genomes Enrichment Analysis

Genes often interact with one another to fulfill specific biological functions. Pathway-based analysis serves as a valuable tool for gaining deeper insights into the biological roles of genes. Kyoto Encyclopedia of Genes and Genomes (KEGG) represents a prominent public database dedicated to pathways. Through pathway enrichment analysis, we identified metabolic pathways or signal transduction pathways that exhibited significant enrichment among the DEGs when compared to the entire genome background.

### 2.7. Quality Property Analysis

Firmness measurements were conducted with minor adjustments following the methodology outlined by Benítez et al. [31] Three fruits were selected, and three measurements per fruit were taken by puncturing the equator using a 3 mm diameter round stainless-steel probe with a flat end and a trigger force of 5 g. The weight of fresh kiwifruits was recorded at 1–4 days intervals during ethylene treatment to determine the weight loss ratio (WLR) of the kiwifruit throughout the treatment period.

For measuring the soluble solids content (SSC), the juice from each fruit was assessed using a temperature-compensated refractometer (SATO SK-102R, Jinan Qiandou Industrial Technology Co., Ltd., Shandong, China) at a constant temperature of 25 °C. The pH of the kiwifruit was determined using a pH meter (E-201-9, Tianjin Xinbode Instrument Co., Ltd., China). The concentration of total soluble sugars (TSS) in the juice was determined by analyzing three slices from each tray using a digital refractometer (DFT-F10V55H23, Shenzhen Liushu Technology Co., Ltd., China).

Respiration rates were measured based on the method described by Xu et al. [32] with slight modifications. Five kiwifruits were placed in a 9.7 L airtight container for 1 h and an infrared gas analyzer (3051P, Hangzhou Lvbo Instrument Co., Ltd., Hangzhou, China) was used to detect CO_2_ production (mmol kg^−1^ h^−1^). Color measurements were conducted following the approach of Benítez et al. [31] with minor adjustments. The CIELAB coordinates (L*, a*, and b*) were determined by assessing three thinly pared sides of each fruit, and the average values of L*, a*, and b* were recorded. The kiwifruit was peeled, then cut into 2 cm thin slices along the equator, and photographs were taken within an airtight photo room (40 × 40 × 40 cm).

### 2.8. Canonical Correlation Analysis

In this study, we adopted canonical correlation analysis (CCA) to explore the potential correlation between quality properties and synthetic genes using transcriptomic data. CCA is a widely utilized analytical method in pattern recognition and machine learning, aiming to identify two weight vectors (u and v) that maximize the correlation coefficient between linear combinations of two sets of random vectors (X and Y) [33]. The objective of this study was to elucidate the relationship between quality properties and genes during the rapid maturation process of instant kiwifruit. To accomplish this, the data was analyzed using Canoco 5.0, and the default parameters provided by the software were utilized for CCA analysis. Through CCA, our investigation aimed to uncover meaningful associations or patterns between quality properties and synthetic genes specific to the rapid maturation process of instant kiwifruit. These findings have the potential to provide valuable insights into the genes and pathways that contribute to the development of desirable fruit-quality traits.

### 2.9. Statistical Analysis

The collected quality parameters were analyzed using Origin (2019). The analysis of variance (ANOVA) was used to assess the significance of differences between cultivars and treatments (*p* < 0.05). A *t*-test was employed to determine the significance of differences between cultivars and treatments. *p* < 0.05 was considered to be statistically significant. Heatmap and principal component analysis (PCA) were conducted. Canoco 5.0 was utilized to import the data for CCA, utilizing the software’s default parameters.

## 3. Results and Discussion

### 3.1. Quality Changes and Principal Component Analysis During Ripening of Ready-to-Eat Kiwifruit

**Figure 1:**
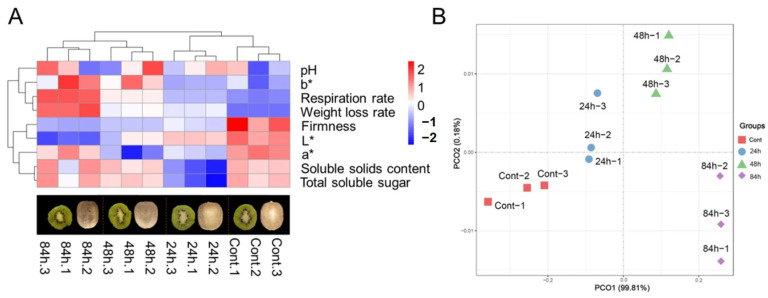
(**A**) The heatmap represents different quality indicators of kiwifruit. The color model is a visual representation of the varying levels of quality. Red signifies high content, while blue represents low content. (**B**) Principal component analysis (PCA) results based on samples at 24 h, 48 h, 84 h, and 0 h. PCA analysis reduces multidimensional data into a few principal components to exhibit similarities and differences among the samples.

Table 1 shows different quality average values based on samples at 0 h, 24 h, 48 h, and 84 h. Figure 1A shows a heatmap that illustrates the temporal changes in various quality attributes of kiwifruit during the ripening process. The heatmap visualization reveals distinct patterns and trends in the ripening process of kiwifruit. Notably, the firmness of the fruit significantly decreased from 297.82 g at 0 h to 27.85 g at 84 h, as depicted by the rich red color. Similar findings on the declining trend of firmness have been reported previously [34,35]. The soluble solids content ranged from 12.74 to 14.00 °Brix, while the total soluble sugar content ranged from 13.15 to 14.37 °Brix. During the early stages of kiwifruit ripening, enhanced respiration increases the energy demand of fruit cells to support growth and development. Soluble sugars serve as primary respiratory substrates and are catabolized to provide energy, leading to a transient decline in SSC and TSS [36]. As the ripening progresses, photosynthetically derived sugars are translocated to the fruit and progressively accumulate, resulting in a rebound of soluble sugar levels. Concurrently, starch reserves in the fruit are hydrolyzed into soluble sugars through enzymatic processes, representing a major contributor to sugar accumulation. This metabolic shift is driven by the dynamic regulation of enzyme activities, which coordinately promote sugar biosynthesis and storage during maturation.

The changes in pH, color parameters, weight loss, and respiration rate during kiwifruit ripening were also analyzed. The pH values remained consistently acidic throughout the ripening process. The lightness (L*) values decreased, indicating a darkening of the fruit surface, while the redness (a*) values showed a decreasing trend, suggesting a diminishing intensity of the red color in the fruit. The yellowness (b*) values exhibited slight fluctuations within the green-yellow range. In terms of weight loss, the kiwifruit samples experienced varying degrees of moisture loss during ripening, with the weight loss rate increasing over time, indicating that water in the fruit migrates to the environment [37]. The respiration rate of kiwifruit progressively increased during ripening, indicating enhanced metabolic activity [38]. The increase in respiration rate marked the initiation of ripening and was synchronized with weight loss, yellowness, and firmness changes.

Figure 1B demonstrates distinct differences among the samples at different time points (0, 24, 48, and 84 h) during the ripening process. A principal component analysis (PCA) was used to analyze the data, and PCO1 and PCO2 explained 99.81% and 0.18% of the overall variance, respectively. At each time point, the quality indicators exhibited significant clustering effects. The exceptionally high variance explained by PC1 (99.81%) demonstrates that all quality indicators undergo highly coordinated changes during kiwifruit ripening. This suggests that the ripening process constitutes a tightly synchronized biological program, characterized by synchronous alterations in key parameters such as firmness, soluble solids content (SSC), and total soluble sugars (TSS). The dominance of PC1 further confirms that differences between ripening stages are primarily driven by time-dependent variations rather than random noise or individual variability. This observation reflects the deterministic nature of ethylene-mediated physiological regulation in climacteric fruit ripening. Conversely, PCO2 (0.18%) contributed only a minimal amount of variance and may lack significant discriminative power in this context.

### 3.2. Transcriptomic Profiles During Kiwifruit Development and Ripening

**Figure 2:**
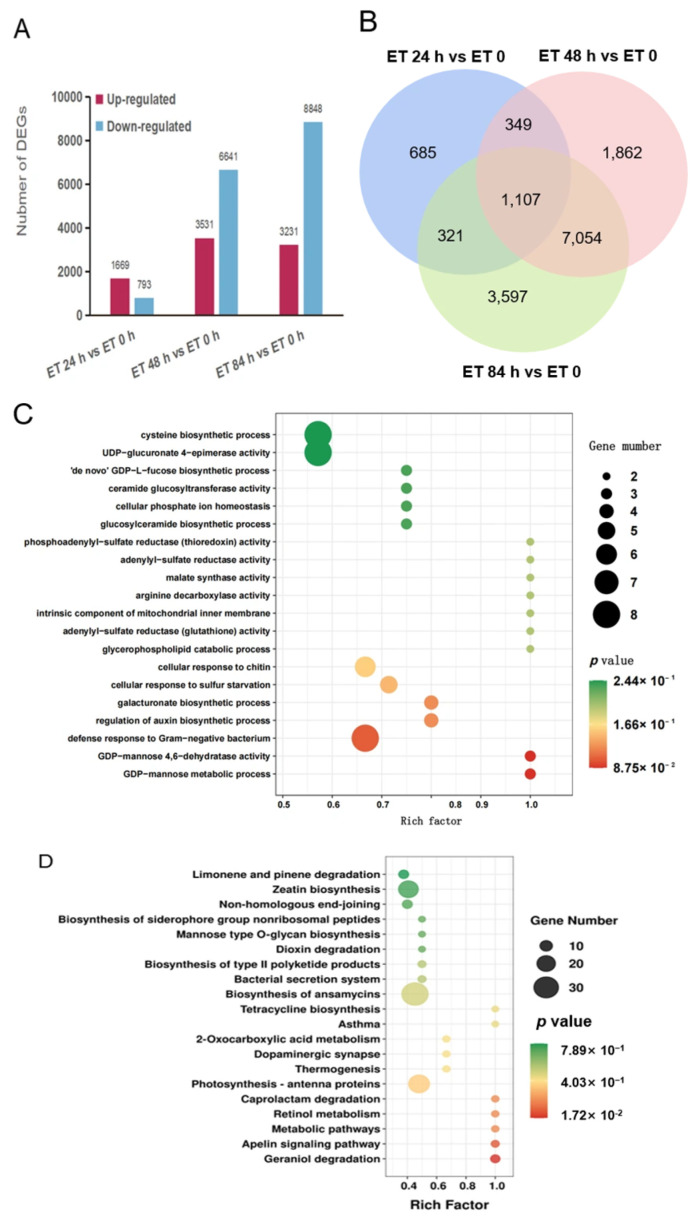
Differentially expressed genes (DEGs). (**A**) Number of DEGs between treated and control samples. DEGs in inoculated samples are shown in red (upregulated) and green (downregulated); (**B**) Venn diagram depicting the number and overlap among DEGs from each time point; (**C**) GO functional enrichment analysis of DEGs; and (**D**) KEGG functional enrichment analysis of DEGs.

All samples exhibited a good sequencing quality and strong correlation with their biological replicates, ensuring the reliability of our transcriptomics data. The differences in transcriptome dynamics across the four developmental stages are presented in Figure 2A,B. The *y*-axis represents the number of genes, while the *x*-axis indicates the different time points. At 24 h, we identified 1669 upregulated and 793 downregulated transcripts with an absolute log2-fold change greater than 1 and a nominal *p*-value less than 0.05. Similarly, at 48 h, 3531 genes were upregulated, and 6841 genes were downregulated compared to the corresponding control. Similarly, at 84 h, we detected 3231 upregulated genes and 8848 downregulated genes. Figure 2B presents a Venn diagram demonstrating the comparison of DEGs at different time points (24, 48, and 84 h) relative to the baseline (0 h).

Furthermore, we performed GO and KEGG pathway enrichment analyses of all DEGs. The GO database, which classifies genes into the categories of molecular function, biological process, and cellular component, provided valuable insights into gene function and classification. The bubble chart representing the enrichment analysis revealed significant biological processes and molecular activities (Figure 2C,D). Twenty enriched GO terms and KEGG pathways (*p*-adj < 0.01) were identified in response to rapid ripening. These processes included the regulation of auxin biosynthesis, galacturonate biosynthesis, cysteine biosynthesis, and so on, indicating the activation of metabolic and defense responses during kiwifruit ripening. Similar findings have been reported in other fruits such as tomatoes, strawberry and bananas [39,40,41]. In tomatoes, cysteine desulfhydrase SlLCD1 is required for endogenous H_2_S generation and regulates fruit ripening [39]. In strawberries, ABA synergizes with auxin (IAA) to coordinate both fruit development and ripening [40]. In bananas, four polygalacturonases (PGs) and three pectin acetylesterases (PAEs), which are involved in homogalacturonan (HG) metabolism, show upregulated expression levels during the later developmental stages. This temporal expression pattern indicates their functional role in regulating fruit expansion [41]. The enriched molecular functions included arginine decarboxylase activity, malate synthase activity, and others. These molecular functions are crucial for various metabolic and enzymatic processes related to kiwifruit ripening [42,43].

During fruit development, the tricarboxylic acid (TCA) cycle plays a vital role by providing reducing equivalents and energy for biosynthetic processes. It involves the incomplete oxidation and subsequent oxidative decomposition of various organic compounds, such as sugars, fatty acids, and amino acids [44]. As part of the TCA cycle, malic acid is produced as a metabolic byproduct. The levels of malic acid have been found to increase significantly during fruit development [45,46]. In kiwifruit, Tang et al. reported the induction of malic enzyme expression within the first seven days after harvest, highlighting the importance of malic acid metabolism in the postharvest ripening of kiwifruit [42]. This study was aimed to investigate the molecular mechanisms underlying the rapid ripening of kiwifruit. We observed a significant upregulation of the gene *Acc26812*, which controls malate synthase activity, during the rapid ripening phase at 84 h, with a fold change of 9. Additionally, arginine decarboxylase is responsible for polyamine biosynthesis, known to participate in various physiological processes, including fruit ripening [43,47]. Previous research has shown that increased arginine decarboxylase transcripts contribute to the ripening process in tomatoes [43]. In our study, we found a significant upregulation of the gene *Acc07097* encoding arginine decarboxylase during the rapid ripening stages, with distinct time points demonstrating fold changes of 15, 10, and 12, indicating a substantial increase in arginine decarboxylase activity. These molecular functions play essential roles in various metabolic and enzymatic processes related to the rapid ripening of kiwifruit.

### 3.3. Canonical Correlation Analysis

**Figure 3:**
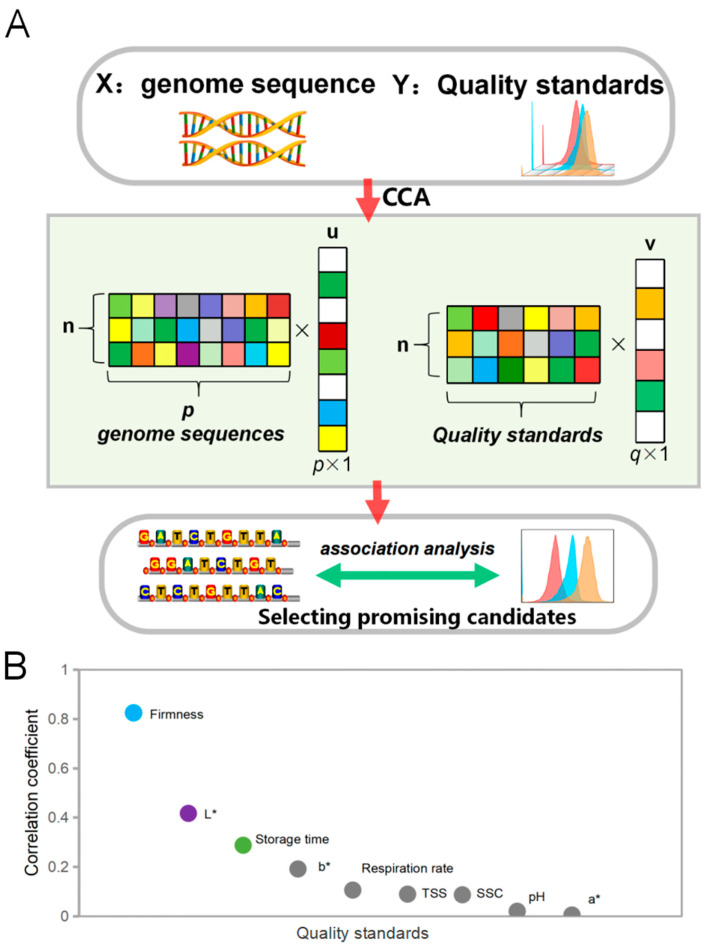
Figure annotation illustrates the results obtained from canonical correlation analysis (CCA) between the matrix of transcriptome data and the matrix of kiwifruit quality indicators. (**A**) Workflow of the analysis. (**B**) Correlation between quality indicators and genes.

Figure 3A provides an overview of the workflow for CCA conducted to integrate the transcriptomic data matrix with the kiwifruit quality indicator matrix. CCA, a structural equation modeling (SEM) technique, was employed to evaluate composite models [48] and establish relationships between gene expression and kiwifruit quality indicators (Figure 3A). Figure 3B illustrates that the transcriptomic data displayed the strongest correlation with the firmness quality indicator. Previous studies have explored the involvement of transcription factors in the ripening process of kiwifruit, as well as those associated with kiwifruit softening following ethylene treatment. However, the present study was the first investigation unveiling the transcriptomic quality indicators as well as their underlying mechanisms related to rapid kiwifruit ripening [19,49]. Therefore, we focused our subsequent analysis on 2000 genes showing significant correlations with variations in firmness. The CCA analysis proved a robust association between the transcriptomic data and the indicators of kiwifruit quality. Among these indicators, the firmness was found to be the most influential factor in shaping the transcriptome, highlighting its critical role in determining kiwifruit quality, suggesting that specific genetic factors may contribute to the observed variations in kiwifruit firmness. The identification of the top 2000 genes exhibiting robust correlations with firmness allows for subsequent in-depth investigations aimed at elucidating the underlying molecular mechanisms responsible for these observed variations.

The identified genes have emerged as promising candidates with a notable significance in regulating kiwifruit quality. An in-depth analysis of their functions and roles could provide valuable insights for advancing kiwifruit breeding programs and augmenting fruit quality. These findings could guide targeted genetic interventions or breeding strategies aimed at achieving desired fruit characteristics, particularly with regard to firmness.

### 3.4. A Comprehensive Gene Ontology and Kyoto Encyclopedia of Genes and Genomes Analysis of Genes Associated with Firmness

**Figure 4:**
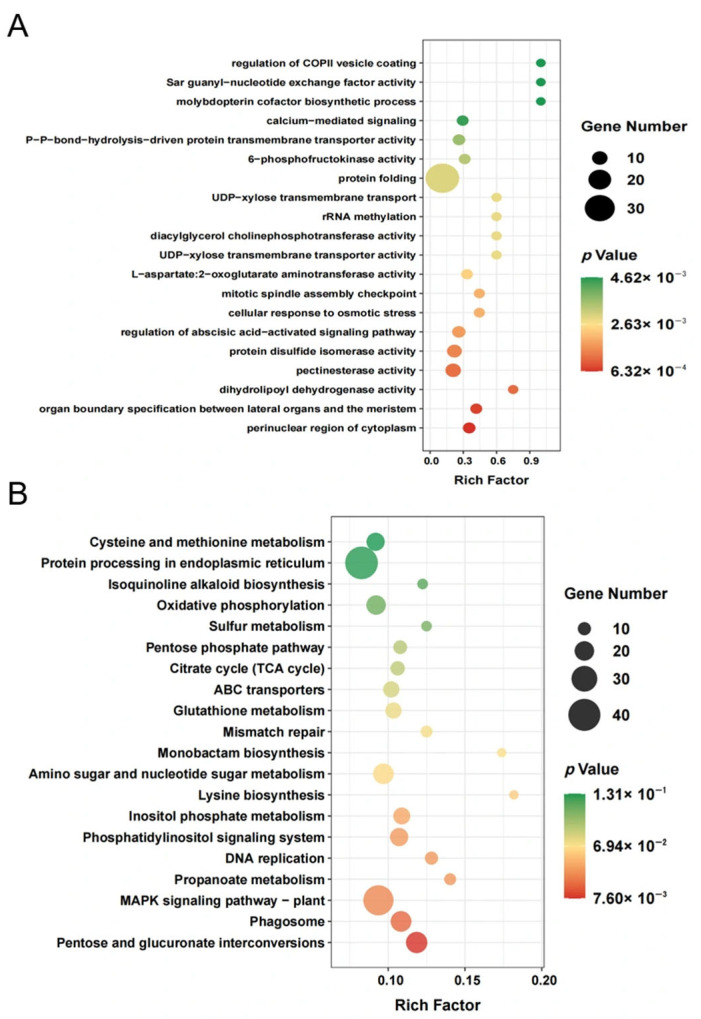
GO enrichment analysis and KEGG pathway enrichment analysis based on the results of CCA. (**A**) GO enrichment; and (**B**) KEGG enrichment.

The analysis of genes associated with firmness was subjected to a GO enrichment analysis, generating a bubble plot to present the results (Figure 4A). This analysis revealed several biological processes and molecular activities linked to the regulation of firmness in kiwifruit. Firstly, regarding cellular localization, the analysis revealed a significant enrichment of genes associated with the perinuclear region of the cytoplasm, suggesting the potential significance of this specific compartment in regulating the firmness. Additionally, the specification of organ boundaries between the lateral organs and the meristem was identified as a significant process associated with the determination of firmness. Furthermore, various enzymatic activities were enriched among the firmness-related genes, including dihydrolipoyl dehydrogenase activity, pectinesterase activity, and protein disulfide isomerase activity. Similar findings have been reported in other fruits such as papaya and plum [50,51], indicating that these enzymatic functions possibly contribute to the biochemical processes involved in the regulation of firmness [52]. The analysis further revealed the enrichment of genes associated with the regulation of specific signaling pathways, including the abscisic-acid-activated signaling pathway and calcium-mediated signaling. Previous studies have demonstrated that reducing the efflux of extracellular Ca^2+^ contributes to the maintenance of cellular vitality and the enhancement in firmness in kiwifruit and apple flesh [53,54]. Moreover, the analysis identified genes associated with cellular responses to osmotic stress and mitotic spindle assembly checkpoint. These fundamental processes play crucial roles in maintaining cellular homeostasis and regulating cell division, which may indirectly influence firmness [55].

Furthermore, several other processes were enriched, including amino acid metabolism (L-aspartate: 2-oxoglutarate aminotransferase activity), carbohydrate metabolism (6-phosphofructokinase activity), membrane transport (UDP-xylose transmembrane transporter activity), and lipid metabolism (diacylglycerol cholinephosphotransferase activity). Additionally, protein folding was found to be enriched. As reported, expansins, identified as cell-wall-localized proteins, play a crucial role in non-enzymic pH-dependent cell wall relaxation [56], possibly through the disruption of hydrogen bonding, underscoring the significance of expansins in preserving the structural integrity of proteins associated with firmness. These findings provide valuable insights into the molecular mechanisms underlying the control of kiwifruit firmness, thereby establishing a solid foundation for subsequent investigations concerning candidate genes and pathways associated with this trait.

The results of the KEGG pathway enrichment analysis for the 2000 genes associated with firmness demonstrated significant enrichments across various metabolic and signaling pathways (Figure 4B). The enriched pathways include a diverse range of biological processes and metabolic networks, including pentose and glucuronate interconversions, phagosome, and the mitogen-activated protein kinase (MAPK) signaling pathway—plant, propanoate metabolism, DNA replication, phosphatidylinositol signaling system, inositol phosphate metabolism, lysine biosynthesis, amino sugar and nucleotide sugar metabolism, monobactam biosynthesis, mismatch repair, glutathione metabolism, ABC transporters, citrate cycle (TCA cycle), pentose phosphate pathway, sulfur metabolism, oxidative phosphorylation, isoquinoline alkaloid biosynthesis, protein processing in the endoplasmic reticulum, and cysteine and methionine metabolism. The enrichment of these pathways suggests the involvement in various biological processes and metabolic interactions associated with the regulation of firmness within the analyzed genes.

The enrichment of specific metabolic and signaling pathways in the analysis of firmness-related genes offers valuable insights into the molecular mechanisms that underlie the regulation of firmness. Notably, the identification of the pentose and glucuronate interconversions pathway indicates the potential involvement of these sugar metabolism pathways in modifying cell wall components, which could influence firmness [57]. Similarly, the enrichment of the amino sugar and nucleotide sugar metabolism pathway points towards the role of sugars in the synthesis of cell wall polysaccharides and their impact on firmness [58]. The MAPK signaling pathway in plant has been well-established to participate in diverse developmental processes and stress responses [59,60]. The observed enrichment of this pathway among the firmness-related genes implies its potential involvement in signaling processes that regulate fruit firmness. The enrichment of the phagosome pathway indicates the potential involvement of phagocytosis-related processes in the determination of firmness, possibly related to the removal or modification of cell wall components [61,62]. The enrichment in the DNA replication pathway suggests that the regulation of cell division and DNA replication processes may have implications for fruit firmness, potentially influencing cell growth and differentiation processes [63]. Additionally, various metabolic pathways associated with organic acid metabolism, including propanoate metabolism and the citrate cycle (TCA cycle), are enriched in firmness-related genes. These pathways contribute to overall cellular metabolism and could have direct or indirect effects on cell wall structure and firmness. Moreover, several other pathways, such as glutathione metabolism, oxidative phosphorylation, and protein processing in the endoplasmic reticulum, are enriched in the analysis. These identified pathways are involved in in crucial cellular processes such as cellular redox homeostasis, energy metabolism, and protein folding, suggesting their potential role in maintaining the structural integrity of firmness-related proteins and overall cellular functions related to the regulation of firmness [64,65]. Overall, the enrichment of these pathways underscores the intricate and multifaceted nature of firmness regulation within the studied organism. Further research and functional analysis of specific genes within these pathways will be instrumental in elucidating the precise mechanisms underlying firmness determination in this specific context.

## 4. Conclusions

This study presents a comparative transcriptome analysis of kiwifruit cultivars treated with ethylene for 84 h at 25 °C. The aim was to identify candidate genes associated with softening and other changes during rapid ripening in ready-to-eat kiwifruit. Specifically, the study identified the gene *ACC26812*, which regulates malate synthase activity, and the gene *ACC07097*, responsible for controlling arginine decarboxylase. These genes exhibited significant enrichment patterns that occurred approximately 3–4 days earlier than the normal ripening process of kiwifruit. Additionally, the top 2000 genes showing strong correlations with firmness were selected to uncover the underlying molecular mechanisms of firmness variations. The results revealed significant enrichments across various metabolic and signaling pathways, including pentose and glucuronate interconversions, phagosome, and the MAPK signaling pathway—plant, among others. This study pioneered the application of the CCA model in fruit-ripening research, thereby transcending the conventional ‘single gene–single phenotype’ analytical paradigm. For the first time, we unveiled the multidimensional regulatory mapping relationships between multi-gene synergistic networks and quality traits, as exemplified by the coordinated impact of the PE-CEL gene cluster on firmness modulation. This methodology establishes a novel toolkit for deciphering the molecular mechanisms underlying complex agronomic traits. Our analysis provides critical gene targets and a theoretical foundation for the targeted regulation of fruit softening, optimization of storage protocols, and establishment of quality standards for ready-to-eat kiwifruit, facilitating the industry’s transition from experience-based harvesting practices to precision-driven quality control systems.

## Figures and Tables

**Table 1:** Different quality average values based on samples at 0 h, 24 h, 48 h, and 84 h.

	ET0	ET24 h	ET48 h	ET84 h	*p*
**Firmness (g)**	297.82	115.52	62.49	27.85	0.007
**Soluble solids content (°Brix)**	13.33	12.74	13.84	14.00	0.385
**Total soluble sugar (°Brix)**	13.82	13.15	14.17	14.37	0.420
**pH**	3.48	3.47	3.51	3.50	0.979
L*	70.03	67.28	65.45	58.38	<0.001
a*	−0.76	−1.26	−1.40	−0.91	0.004
b*	26.67	27.46	30.96	31.87	0.019
**Respiration rate (mg·Kg ^−1^·h ^−1^)**	6.15	10.11	16.72	28.17	<0.001
**Weight loss rate (%)**	0.00	0.33	0.40	0.91	<0.001

## Data Availability

The original contributions presented in the study are included in the article. Further inquiries can be directed to the corresponding author.

## References

[1] Ma T., Lan T., Geng T., Ju Y., Cheng G., Que Z., Gao G., Fang Y., Sun X. (2019). Nutritional properties and biological activities of kiwifruit (Actinidia) and kiwifruit products under simulated gastrointestinal in vitro digestion. Food Nutr. Res..

[2] Ferguson A., Stanley R. (2003). Kiwifruit, Encyclopedia of Food Sciences and Nutrition.

[3] Guo J., Yuan Y., Dou P., Yue T. (2017). Multivariate statistical analysis of the polyphenolic constituents in kiwifruit juices to trace fruit varieties and geographical origins. Food Chem..

[4] Ma T., Sun X., Zhao J., You Y., Lei Y., Gao G., Zhan J. (2017). Nutrient compositions and antioxidant capacity of kiwifruit (*Actinidia*) and their relationship with flesh color and commercial value. Food Chem..

[5] Li J., Li Y., Wu H., Naraginti S., Wu Y. (2021). Facile synthesis of ZnO nanoparticles by Actinidia deliciosa fruit peel extract: Bactericidal, anticancer and detoxification properties. Environ. Res..

[6] Choi H.R., Tilahun S., Park D.S., Lee Y.M., Choi J.H., Baek M.W., Jeong C.S. (2019). Harvest time affects quality and storability of kiwifruit (*Actinidia* spp.) Cultivars during long-term cool storage. Sci. Hortic..

[7] Bakhshipour A. (2023). A data fusion approach for nondestructive tracking of the ripening process and quality attributes of green Hayward kiwifruit using artificial olfaction and proximal hyperspectral imaging techniques. Food Sci. Nutr..

[8] Mao J., Gao Z., Lin M., Zhang X., Ning X., Gong X., Lu Y., Chen L., Wang X. (2023). Targeted multi-platform metabolome analysis and enzyme activity analysis of kiwifruit during postharvest ripening. Front. Plant Sci..

[9] Peng Z., Liu G., Li H., Wang Y., Gao H., Jemrić T., Fu D. (2022). Molecular and Genetic Events Determining the Softening of Fleshy Fruits: A Comprehensive Review. Int. J. Mol. Sci..

[10] Atkinson R.G., Gunaseelan K., Wang M.Y., Luo L., Wang T., Norling C.L., Johnston S.L., Maddumage R., Schröder R., Schaffer R.J. (2011). Dissecting the role of climacteric ethylene in kiwifruit (*Actinidia chinensis*) ripening using a 1-aminocyclopropane-1-carboxylic acid oxidase knockdown line. J. Exp. Bot..

[11] Fan X., Shu C., Zhao K., Wang X., Cao J., Jiang W. (2018). Regulation of apricot ripening and softening process during shelf life by post-storage treatments of exogenous ethylene and 1-methylcyclopropene. Sci. Hortic..

[12] Hu Z.L., Deng L., Chen X.Q., Wang P.Q., Chen G.P. (2010). Co-suppression of the *EIN2*-homology gene *LeEIN2* inhibits fruit ripening and reduces ethylene sensitivity in tomato. Russ. J. Plant Physiol..

[13] Kim J., Lee J.G., Lim S., Lee E.J. (2023). A comparison of physicochemical and ripening characteristics of golden-fleshed ‘Haegeum’ and green-fleshed ‘Hayward’ kiwifruit during storage at 0 °C and ripening at 25 °C. Postharvest Biol. Technol..

[14] Miao H., Zhang J., Zheng Y., Jia C., Hu Y., Wang J., Zhang J., Sun P., Jin Z., Zhou Y. (2025). Shaping the future of bananas: Advancing genetic trait regulation and breeding in the postgenomics era. Hortic. Res..

[15] Su W., Chen X., Wei W., Kou Y., Deng C., Lin H., Chen Y., Xu Q., Wu L., Zhu C. (2025). Occurrence of White Flesh Color and Refreshing Flavor Following *Phytoene Synthase 2A* Gene Variation in Loquat Fruit. J. Agric. Food Chem..

[16] Slugina M.A., Efremov G.I., Shchennikova A.V., Kochieva E.Z. (2021). Characterization of *RIN* Isoforms and Their Expression in Tomato Fruit Ripening. Cells.

[17] Tang Y., Yan Y., Tie W., Ye X., Zeng L., Zeng L., Yang J., Xu B., Li M., Wang Y. (2023). Transcriptional regulation of *MbACO2*-mediated ethylene synthesis during postharvest banana ripening. Postharvest Biol. Technol..

[18] Zeng J., Jiang G., Liang H., Yan H., Kong X., Duan X., Li Z. (2023). Histone demethylase MaJMJ15 is involved in the regulation of postharvest banana fruit ripening. Food Chem..

[19] Huang G., Qu Y., Li T., Yuan H., Wang A., Tan D. (2018). Comparative Transcriptome Analysis of *Actinidia arguta* Fruits Reveals the Involvement of Various Transcription Factors in Ripening. Hortic. Plant J..

[20] Wang R., Shu P., Zhang C., Zhang J., Chen Y., Zhang Y., Du K., Xie Y., Li M., Ma T. (2022). Integrative analyses of metabolome and genome-wide transcriptome reveal the regulatory network governing flavor formation in kiwifruit (*Actinidia chinensis*). N. Phytol..

[21] Shan N., Zhang Y., Xu Y., Yuan X., Wan C., Chen C., Chen J., Gan Z. (2022). Ethylene-induced potassium transporter *AcKUP2* gene is involved in kiwifruit postharvest ripening. BMC Plant Biol..

[22] Zhang Q.-Y., Ge J., Liu X.-C., Wang W.-Q., Liu X.-F., Yin X.-R. (2022). Consensus co-expression network analysis identifies AdZAT5 regulating pectin degradation in ripening kiwifruit. J. Adv. Res..

[23] Chen Y., Shu P., Wang R., Du X., Xie Y., Du K., Deng H., Li M., Zhang Y., Grierson D. (2021). Ethylene response factor AcERF91 affects ascorbate metabolism via regulation of GDP-galactose phosphorylase encoding gene (AcGGP3) in kiwifruit. Plant Sci..

[24] Shin M.H., Muneer S., Kim Y.-H., Lee J.J., Bae D.W., Kwack Y.-B., Kumarihami H.M.P.C., Kim J.G. (2020). Proteomic analysis reveals dynamic regulation of fruit ripening in response to exogenous ethylene in kiwifruit cultivars. Hortic. Environ. Biotechnol..

[25] Nieuwenhuizen N.J., Chen X., Pellan M., Zhang L., Guo L., Laing W.A., Schaffer R.J., Atkinson R.G., Allan A.C. (2021). Regulation of wound ethylene biosynthesis by NAC transcription factors in kiwifruit. BMC Plant Biol..

[26] Ampomah-Dwamena C., Thrimawithana A.H., Dejnoprat S., Lewis D., Espley R.V., Allan A.C. (2019). A kiwifruit (*Actinidia deliciosa*) R2R3-MYB transcription factor modulates chlorophyll and carotenoid accumulation. N. Phytol..

[27] Babaei-Rad S., Mumivand H., Mollaei S., Khadivi A. (2025). Postharvest UV-B and UV-C treatments combined with fermentation enhance the quality characteristics of *Capparis spinosa* L. fruit, improving total phenols, flavonoids, anthocyanins, phenolic acids, and antioxidant activity. Food Chem..

[28] Zheng Y., Lin Y., Wen H., Sang Y., Lin M., Fan Z., Wang H., Chen Y., Lin Y., Lin H. (2025). The role of respiration metabolism in dicyclohexylcarbodiimide and disodium succinate regulating the pulp breakdown occurrence of fresh longan (*Dimocarpus longan* Lour.) during storage. Food Chem. X.

[29] Wang N., Liu W., Zhang T., Jiang S., Xu H., Wang Y., Zhang Z., Wang C., Chen X. (2018). Transcriptomic Analysis of Red-Fleshed Apples Reveals the Novel Role of MdWRKY11 in Flavonoid and Anthocyanin Biosynthesis. J. Agric. Food Chem..

[30] Zhao C., Cheng L., Guo Y., Hui W., Niu J., Song S. (2024). An integrated quality, physiological and transcriptomic analysis reveals mechanisms of kiwifruit response to postharvest transport vibrational stress. Plant Physiol. Biochem..

[31] Benitez S., Achaerandio I., Sepulcre F., Pujola M. (2013). Aloe vera based edible coatings improve the quality of minimally processed ‘Hayward’ kiwifruit. Postharvest Biol. Technol..

[32] Xu F., Liu S., Liu Y., Xu J., Liu T., Dong S. (2019). Effectiveness of lysozyme coatings and 1-MCP treatments on storage and preservation of kiwifruit. Food Chem..

[33] Zhao S., Ding Y., Liu X., Su X. (2022). HKAM-MKM: A hybrid kernel alignment maximization-based multiple kernel model for identifying DNA-binding proteins. Comput. Biol. Med..

[34] Chen Y., Hu X., Shi Q., Lu Y., Yan J., Wu D.-T., Qin W. (2023). Changes in the Fruit Quality, Phenolic Compounds, and Antioxidant Potential of Red-Fleshed Kiwifruit during Postharvest Ripening. Foods.

[35] Huang W., Wang Z., Zhang Q., Feng S., Burdon J., Zhong C. (2022). Maturity, Ripening and Quality of ‘Donghong’ Kiwifruit Evaluated by the Kiwi-Meter^TM^. Horticulturae.

[36] Giovannoni J.J. (2004). Genetic regulation of fruit development and ripening. Plant Cell.

[37] Zhao X., Meng X., Li W., Cheng R., Wu H., Liu P., Ma M. (2021). Effect of hydrogen-rich water and slightly acidic electrolyzed water treatments on storage and preservation of fresh-cut kiwifruit. J. Food Meas. Charact..

[38] Yan H., Wang R., Ji N., Cao S., Ma C., Li J., Wang G., Huang Y., Lei J., Ba L. (2022). Preparation, Shelf, and Eating Quality of Ready-to-Eat “Guichang” Kiwifruit: Regulation by Ethylene and 1-MCP. Front. Chem..

[39] Hu K.-D., Zhang X.-Y., Yao G.-F., Rong Y.-L., Ding C., Tang J., Yang F., Huang Z.-Q., Xu Z.-M., Chen X.-Y. (2020). A nuclear-localized cysteine desulfhydrase plays a role in fruit ripening in tomato. Hortic. Res..

[40] Li T., Dai Z., Zeng B., Li J., Ouyang J., Kang L., Wang W., Jia W. (2022). Autocatalytic biosynthesis of abscisic acid and its synergistic action with auxin to regulate strawberry fruit ripening. Hortic. Res..

[41] Ning T., Chen C., Yi G., Chen H., Liu Y., Fan Y., Liu J., Chen S., Wei S., Li Z. (2021). Changes in Homogalacturonan Metabolism in Banana Peel during Fruit Development and Ripening. Int. J. Mol. Sci..

[42] Tang W., Zheng Y., Dong J., Yu J., Yue J., Liu F., Guo X., Huang S., Wisniewski M., Sun J. (2016). Comprehensive Transcriptome Profiling Reveals Long Noncoding RNA Expression and Alternative Splicing Regulation during Fruit Development and Ripening in Kiwifruit (*Actinidia chinensis*). Front. Plant Sci..

[43] Tsaniklidis G., Charova S.N., Fanourakis D., Tsafouros A., Nikoloudakis N., Goumenaki E., Tsantili E., Roussos P.A., Spiliopoulos I.K., Paschalidis K.A. (2021). The role of temperature in mediating postharvest polyamine homeostasis in tomato fruit. Postharvest Biol. Technol..

[44] Omini J.J., Krassovskaya I., Obata T. (2022). Malate Dehydrogenase-Citrate Synthase Multienzyme Complex Dynamics Is Affected By TCA Cycle Flux In Living Yeast Cells. FASEB J..

[45] Jia D., Xu Z., Chen L., Huang Q., Huang C., Tao J., Qu X., Xu X. (2023). Analysis of organic acid metabolism reveals citric acid and malic acid play major roles in determining acid quality during the development of kiwifruit (*Actinidia eriantha*). J. Sci. Food Agric..

[46] Sun X., Han G., Meng Z., Lin L., Sui N. (2019). Roles of malic enzymes in plant development and stress responses. Plant Signal. Behav..

[47] Rastogi R., Davies P.J. (1991). Polyamine Metabolism in Ripening Tomato Fruit: II. Polyamine Metabolism and Synthesis in Relation to Enhanced Putrescine Content and Storage Life of a/c Tomato Fruit. Plant Physiol..

[48] Hubona G.S., Schuberth F., Henseler J. (2021). A clarification of Confirmatory Composite Analysis (CCA). Int. J. Inf. Manag..

[49] Choi H., Baek M., Jeong C., Tilahun S. (2022). Comparative Transcriptome Analysis of Softening and Ripening-related Genes in Kiwifruit Cultivars Treated with Ethylene. Curr. Issues Mol. Biol..

[50] He M., Wu Y., Wang Y., Hong M., Li T., Deng T., Jiang Y. (2022). Valeric acid suppresses cell wall polysaccharides disassembly to maintain fruit firmness of harvested ‘Waizuili’ plum (*Prunus salicina Lindl*). Sci. Hortic..

[51] Zerpa-Catanho D., Esquivel P., Mora-Newcomer E., Saenz M.V., Herrera R., Jimenez V.M. (2017). Transcription analysis of softening-related genes during postharvest of papaya fruit (*Carica papaya* L. ‘Pococi’ hybrid). Postharvest Biol. Technol..

[52] Soares C.G., do Prado S.B.R., Andrade S.C.S., Fabi J.P. (2021). Systems Biology Applied to the Study of Papaya Fruit Ripening: The Influence of Ethylene on Pulp Softening. Cells.

[53] Huang Y., Qiu L., Wang Y., Yuan Y., Qu H. (2020). Ca^2+^ efflux is negatively correlated with apple firmness. Sci. Hortic..

[54] Polychroniadou C., Karagiannis E., Michailidis M., Adamakis I.-D.S., Ganopoulos I., Tanou G., Bazakos C., Molassiotis A. (2022). Identification of genes and metabolic pathways involved in wounding-induced kiwifruit ripening. Plant Physiol. Biochem..

[55] Gerlee P., Basanta D., Anderson A.R.A. (2017). The Influence of Cellular Characteristics on the Evolution of Shape Homeostasis. Artif. Life.

[56] Burdon J., Martin P., Ireland H., Schaffer R., McAtee P., Boldingh H., Nardozza S. (2021). Transcriptomic analysis reveals differences in fruit maturation between two kiwifruit cultivars. Sci. Hortic..

[57] Wang S., Liu C., Su X., Chen L., Zhu Z. (2023). Transcriptome analysis reveals key metabolic pathways and gene expression involving in cell wall polysaccharides-disassembling and postharvest fruit softening in custard apple (*Annona squamosa* L.). Int. J. Biol. Macromol..

[58] Schwerdt J., Qiu H., Shirley N., Little A., Bulone V. (2021). Phylogenomic Analyses of Nucleotide-Sugar Biosynthetic and Interconverting Enzymes Illuminate Cell Wall Composition in Fungi. mBio.

[59] Manna M., Rengasamy B., Sinha A.K. (2023). Revisiting the role of MAPK signalling pathway in plants and its manipulation for crop improvement. Plant Cell Environ..

[60] Sun T., Zhang J., Zhang Q., Li X., Li M., Yang Y., Zhou J., Wei Q., Zhou B. (2022). Exogenous application of acetic acid enhances drought tolerance by influencing the MAPK signaling pathway induced by ABA and JA in apple plants. Tree Physiol..

[61] Chiplunkar S.S., Silva C.A., Bermudez L.E., Danelishvili L. (2019). Characterization of membrane vesicles released by Mycobacterium avium in response to environment mimicking the macrophage phagosome. Futur. Microbiol..

[62] Ojkic N., Lopez-Garrido J., Pogliano K., Endres R.G. (2016). Cell-wall remodeling drives engulfment during *Bacillus subtilis* porulation. Elife.

[63] Tahmasebi A., Ashrafi-Dehkordi E., Shahriari A.G., Mazloomi S.M., Ebrahimie E. (2019). Integrative meta-analysis of transcriptomic responses to abiotic stress in cotton. Prog. Biophys. Mol. Biol..

[64] Zhu Y., Qiu W., He X., Wu L., Bi D., Deng Z., He Z., Wu C., Zhuo R. (2022). Integrative analysis of transcriptome and proteome provides insights into adaptation to cadmium stress in *Sedum plumbizincicola*. Ecotoxicol. Environ. Saf..

[65] Wang Q., Zhang D., Liu J., Shang B., Duan X., Sun H. (2022). Storage Drives Alterations of Proteomic and Protein Structural Properties in Rice (*Oryza sativa* L.). Foods.

